# Latitudinal variation in the woody species diversity and population structure of *Lannea microcarpa* Engl. and K. Krause in Burkina Faso

**DOI:** 10.1016/j.heliyon.2022.e09625

**Published:** 2022-06-04

**Authors:** Béatrice Tinguéri, Kangbéni Dimobe, Benjamin Lankoandé, Joseph Issaka Boussim, Amadé Ouédraogo

**Affiliations:** a Laboratoire de Biologie et Ecologie Végétales, UFR Sciences de la Vie et de la Terre, Université Joseph Ki-Zerbo, 03 Po Box 7021, Ouagadougou 03, Burkina Faso; b Institut des Sciences de l’Environnement et du Développement Rural (ISEDR), Université de Dédougou, BP 176, Dédougou, Burkina Faso; c Université de Fada N'Gourma, Po Box 54, Fada N'Gourma, Burkina Faso

**Keywords:** African grape tree, Burkina Faso, Diversity, Phytogeographical gradient, Semi-arid zone, Size class distribution

## Abstract

Latitude is source of variations of plant species diversity and stand structure. This study aimed to characterize the populations of the African grape tree, *Lannea microcarpa*, in its range in Burkina Faso. Data were collected according to oriented sampling scheme, based on the presence of the species. Overall, 140 inventory plots of 1000 m^2^ each were considered across the sub-Sahelian, north-Sudanian and south-Sudanian phytogeographical zones. Tree diameter at breast height (DBH) and total height of *L. microcarpa* individuals were measured and all woody species in each plot were recorded. A comparative analysis was made between zones for woody flora diversity associated with *L. microcarpa*, dendrometric and stand structure parameters. The results revealed a total woody flora richness of 109 species. Correlogram analysis revealed that species associated with *L. microcarpa* differed between phytogeographical zones. Ten species showed a probability of association with *L. microcarpa*. The density of *L. microcarpa* populations and the floristic diversity of its habitats decreased significantly (p < 0.001) from sub-Sahelian to south-Sudanian zones. The diameter classes’ structure in the three phytogeographical zones revealed unstable populations with a predominance of intermediate classes compared to extreme classes. The height structure of juveniles is marked by a poor transition between the different classes, showing unpredictable regeneration of *L. microcarpa* in the three phytogeographical zones. The results of this study draw attention to the need to adopt conservation measures for the species. In this sense, the data on the comparative demographic characteristics can serve as a basis for the implementing of adequate management strategies of natural populations of the species across its distribution range in Burkina Faso.

## Highlights

• Latitudinal variation influence the population structure of *Lannea macrocarpa* and woody species associated to its stands.
• The species experiences difficulties in recruitment and transition between the different height classes in the study sites.
• Additional actions will help to study in depth the human disturbance regimes on its population structure.

## Teaser

African grape tree, Phytogeographical gradient, Diversity, Size class distribution, Semi-arid zone, Burkina Faso.

## 1 Introduction

West African savannah ecosystems are undergoing drastic changes in the composition and structure of their vegetation due to the impact of human activities and climatic pejoration [1]. Indeed, with the deterioration of climatic conditions and population growth in recent decades, savanna ecosystems are being degraded, compromising the supply of natural resources [2]. Several species are being overexploited, especially those that provide Non- Timber Forest Products (NTFPs) and have a high socio-economic potential. This is the case of wild fruit trees such as shea (*Vitellaria paradoxa* C. F. Gaertn), African locust bean (*Parkia biglobosa,* Jacq. Dong), and desert date palm (*Balanites aegyptiaca* L. Delile), which are important sources of income for vulnerable households, thus reducing their poverty [3, 4]. Shrestha et al. [5], has shown that in recent decades, demand for NTFPs has increased worldwide and particularly in developing countries due to high population growth. This increasing demand can lead to a change in the population structure of NTFPs providing-species, resulting in the scarcity or absence of seedlings [6] to ensure the regeneration [6, 7]. Like these edible fruit trees, *Lannea microcarpa* Engl. et K. Krause (*L. macrocarpa*), called African grape tree, is not immune to this overexploitation because it is highly valued by local populations as a result of the various ecosystem services it provides.

*L. microcarpa* is known for its food uses. The fruits ripen during the lean season, which makes it particularly important in times of food shortage [8]. Also, it is spared with other species during agricultural clearings making it a major species in agroforestry parks. Because of its crucial importance for local populations and to ensure its sustainable management in order to promote its domestication, several ecological and ethnobotanical studies have been conducted on the species in West Africa. Indeed, these studies have focused on different uses of the species [9, 10], its fruit production according to land use types and climatic gradient, the nutritional composition of its fruits [11, 12, 13], its natural seed regeneration and vegetative propagation abilities [14, 15] and its population structure according to land use types and habitat characterization [11, 16]. However, there is no up-to-date scientific information on the demographic variability of the species along the latitudinal gradient of its distribution range in Burkina Faso taking into account the three phytogeographical zones. Understanding such demographic patterns is an important step to guide conservation priorities. However, phytogeographical alterations are factors that modify the diversity of woody species and latitudinal variations influence the diversity and species populations structure through climate and anthropogenic disturbances [17, 18]. Thus, *L. microcarpa* structural characteristics are expected to be correlated positively with increasing rainfall. Also, knowing that the survival of a plant depends on the health of the ecosystem that shelters it [19], it is necessary to assess the diversity of the woody flora in the habitats of *L. microcarpa* and to identify the species closely associated with it. This approach, which is based on forest inventory and floristic data, will make it possible to understand the spatial dynamics of the grape tree within its range in Burkina Faso and to better understand its level of vulnerability in order to direct conservation and sustainable management strategies.

The main objective of this study is to characterize the current status of *L. microcarpa* populations along a phytogeographic gradient in Burkina Faso in order to provide basic tools for its sustainable management. Specifically, the study aims to: (i) describe the diversity of woody species associated with *L. microcarpa* in its natural habitat and (ii) determine the status of *L. microcarpa* natural populations across three phytogeographical zones in Burkina Faso. Considering that the ecological status of a species’ stands varies along a latitudinal gradient, we hypothesized that the habitat diversity and the structural parameters of the species vary according to the phytogeographical zones in Burkina Faso.

## 2 Material and methods

### 2.1 Study area

**Figure 1:**
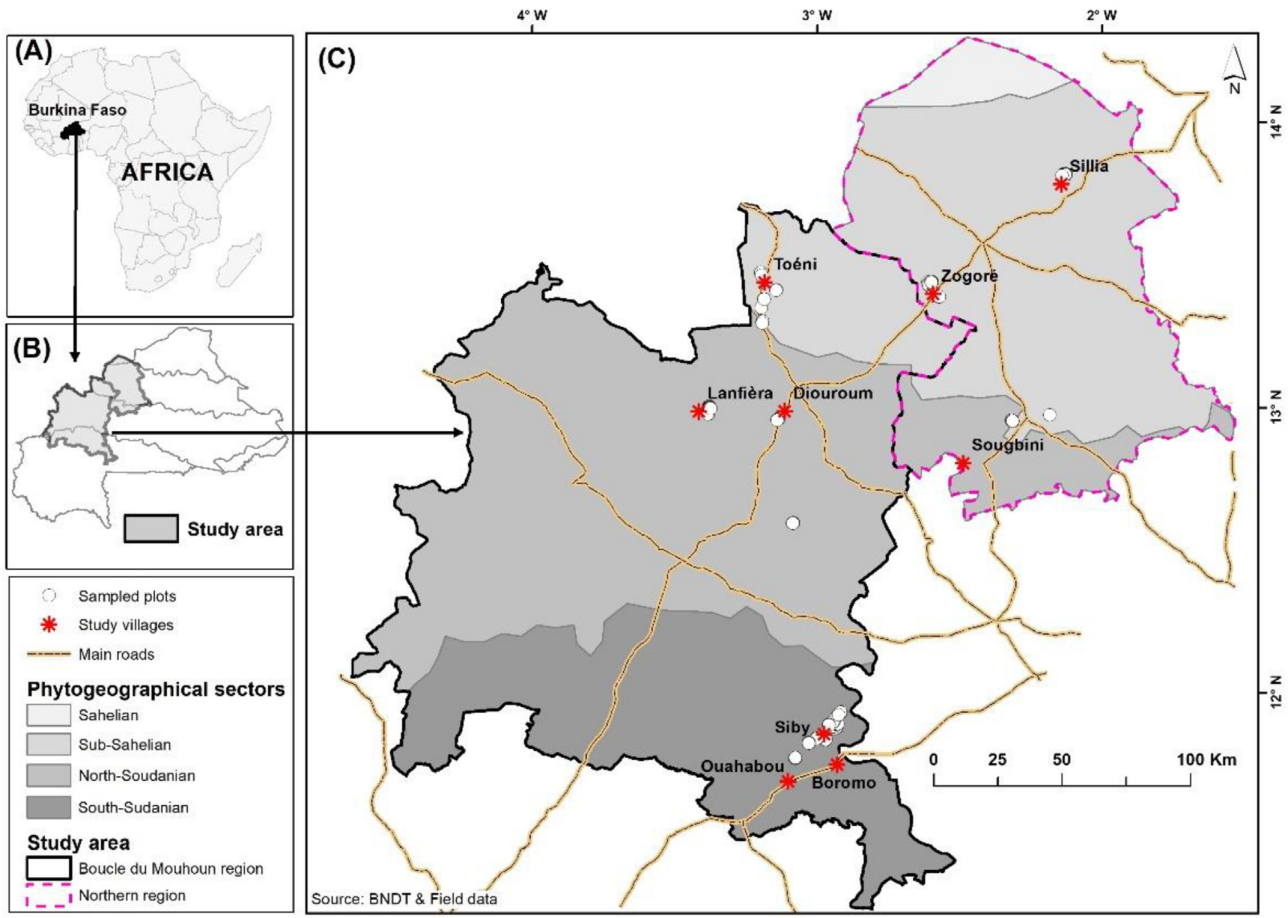
Maps showing (A) the location of Burkina Faso in Africa, (B) the location of the study area in Burkina Faso and (C) the location of the study villages and sampled plots in the study area.

The study was carried out across three phytogeographical zones of Burkina Faso [20]. These are the sub-Sahelian (between latitudes 12°55′N and 14°10′N), the north-Sudanian (between latitudes 12°25′N and 12°55′N) and the south-Sudanian zones (between latitudes 11°15′N and 12°25′N), following a north-south latitudinal gradient (Figure 1). The main stands of *L. microcarpa* were identified after field exploration of the species' preferred habitats across the three zones, which integrate the influence of anthropogenic, ecological, and climatic factors on the species. A total of nine sites, three per phytogeographical zone, were chosen based on the presence of *L. microcarpa* populations. The climatic and ecological characteristics of the phytogeographical areas hosting these sites are described in Table 1.

The most common soil types in the sub-Sahelian, north-Sudanian and south-Sudanian zones are eroded tropical ferruginous soils, poorly developed soils, tropical soils with little or no leaching and hydromorphic soils with pseudo-gley, respectively [21, 22].

**Table 1:** Climatic and vegetation characteristics of the study area.

Phytogeographical zones	Study sites	Mean rainfall (mm)	Rainy days	Mean temperature (°C)	Dominant vegetation	Main species
Sub-Sahelian	Silia	720	40–50	30	Steppe and thickets	*Senegalia laeta; Vachellia nilotica; Senegalia senegal; Balanites aegyptiaca,*
	Zogoré					
	Toéni					
North- Sudanian	DiouroumLanfiéra	835	40–70	29	Shrub and tree savannas	*Faidherbia albida; Lannea microcarpa; Tamarindus indica; Vitellaria paradoxa*
	Sougbini					
South-Sudanian	Boromo Ouahabou	1042	70–90	27	Mosaic of savannas and gallery forests	*Anogeisus leiocarpa; Burkea africana; Isoberlinia doka; Pteleopsis suberosa*
	Siby					

Rainfall and temperature (Data from the National Meteorological Agency of Burkina Faso from 1989-2019).

Vegetation types and main species [23].

The human population of the study area is predominantly made up of Mossi, San, Bwaba and Fulani. The main practiced activities are agriculture, livestock, forest products harvesting and trade.

### 2.2 Description of the study species

**Photo1:**
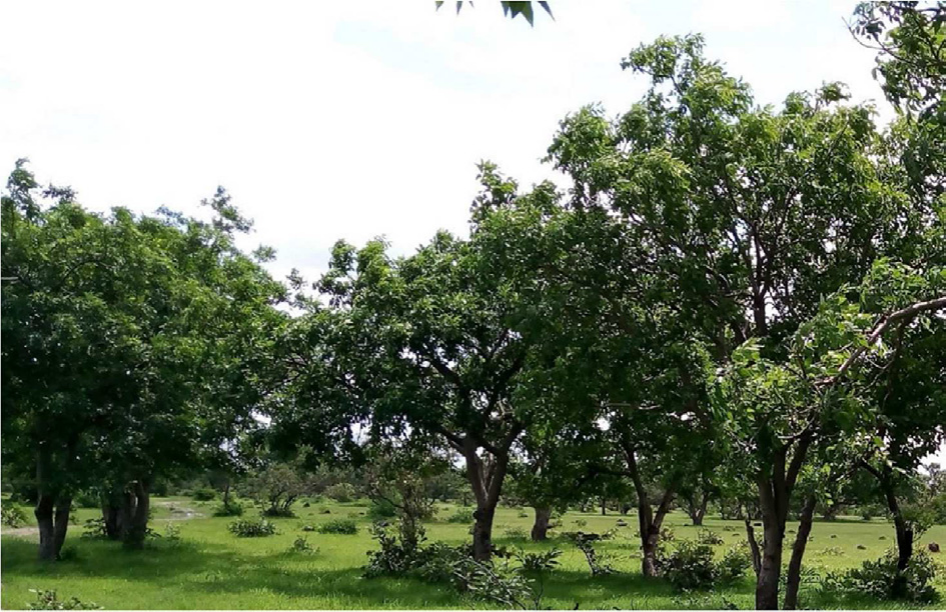
Stand of *L. microcarpa* in the south-Sudanian zone, site of Boromo (Photo Tingueri, 2019).

**Photo 2:**
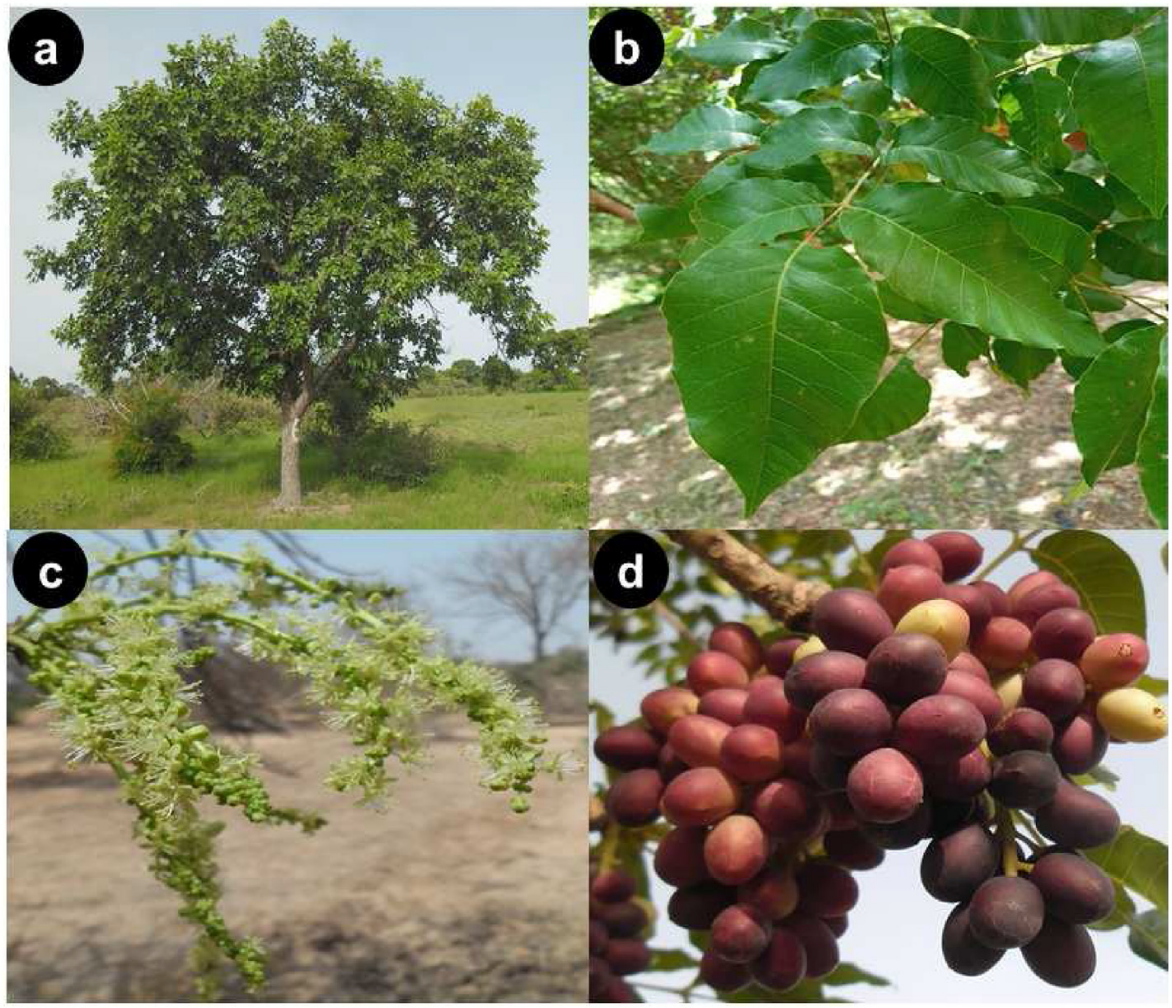
*Lannea microcarpa* tree (a), its leaves (**b**), flowers (**c**) and fruits (**d**) (Photo Tingueri, 2019).

*Lannea microcarpa* is a dioecious tree species belonging to the Anacardiaceae family. The northern limit of its habitat is the Sahelo-Sudanian zone (500–900 mm) and the southern limit is the Guinean zone (>1,100 mm) [11] The species occurs in the Sahelo-Sudanian and Sudanian savannas, from Senegal to Cameroon [24] where it forms extensive populations (Photo 1). *Lannea microcarpa* tree can reach up to 15 m in height and a DBH of up to 70 cm (Photo 2a). It has alternate odd-pinnately compound leaves (Photo 2b). The inflorescence is a terminal raceme with small and inconspicuous yellowish flowers (Photo 2c). Fruits are ellipsoidal drupes about 1 cm long, hairless, turning from green to dark purple at maturity (Photo 2d). Foliage begins shortly after flowering at the end of the dry season and fruiting coincides with the onset of the rainy seasons [24]. Seeds have a high lipid content (more than 60%) and are highly stable to oxidation, hence their use as frying oil [25]. According to the Agency for the Promotion of Non-Timber Forest Products of Burkina Faso (APFNL), *L. microcarpa* has a high socio-economic value. Indeed, its fruits are intensively traded during the production period between April and July.

### 2.3 Sampling and data collection

Data were collected following a stratified and directed sampling scheme based on the presence of *L. microcarpa* populations, in nine sites distributed across three phytogeographical zones (i.e. 3 sites per phytogeographical zone). The selection of sites was made by taking into account their accessibility and the availability of the species. A total of 140 rectangular plots of 1000 m^2^ (50 m × 20 m) were established in the natural stands of *L. microcarpa*, distributed in 45 plots in the south- Sudanian, 48 in the north-Sudanian and 47 in the sub-Sahelian. In each plot, floristic inventories were carried out to record the woody flora associated with *L. microcarpa*. Furthermore, forest inventories were conducted to assess the population structure of the species throughout the measurements of the (i) diameter at breast height (DBH), (ii) total height of its individual trees, and (iii) regeneration of its individuals. The stem diameter of all *L. microcarpa* trees (dbh ≥ 5 cm) were measured using a diameter tape. Their total height were measured using a clinometer. To characterize the regeneration of the species, two sub-plots of 25 m^2^ (5 m × 5 m) were installed in each rectangular plot with a focus on *L. microcarpa* regeneration. In each sub-plot the height of juvenile individuals (DBH< 5 cm) of the species was measured and classified into five height classes of amplitude 0.5 m.

### 2.4 Data analysis

#### 2.4.1 Ecological parameters of habitats and floristic diversity of *Lannea microcarpa* stands

The composition of woody species associated with *L. microcarpa* was determined by investigating their co-occurrence in its stands. Co-occurrence patterns of species were assessed by calculating the frequency of plots where each species is jointly recorded with *L. microcarpa* [26]. A hierarchical classification was used to classify species according to the degree of co-occurrence. The results were presented using a correlogram which is an advanced graphical tool [26]. To reduce the number of variables in the figure, only 18 species recorded in at least 30 plots in each phytogeographical zone were considered for the correlogram analysis.

To compare and better reflect the floristic diversity in the three phytogeographical zones, taxonomic diversity, species richness, evenness index of Pielou and Shannon–Wiener (*H’*) diversity index were computed following Magurran [27]. Moreover, to quantify the similarity among the populations of the species, we computed the Jaccard similarity index (Cj) as follow:

(1) Cj=j/(a+b−j)

where *j* represents the number of species common to two sites A and B, *a* is the number of species at site A and *b* is the number of species at site B.

#### 2.4.2 Spatial distribution of *Lannea microcarpa*

The spatial distribution pattern of *L. microcarpa* in the three phytogeographical zones was characterized by computing the Green index (GI) [28] according to the following formula:

(2) GI=(IB−1)/(n−1)

with *n* the total number of adult individuals *IB = σ*^*2*^*/N* which is the Blackman dispersion index while *σ*^*2*^ is the variance and *N* the mean value of tree density. The GI values vary from -1 to +1 with the value 1 indicating an extremely aggregated distribution of trees.

#### 2.4.3 Dendrometric parameters of *Lannea microcarpa* populations

The following dendrometric parameters were calculated per phytogeographical zone:

• Density (individuals/ha) of trees and juveniles: it was calculated as the average number of trees or juveniles per hectare
• Basal area (G, in m^2^/ha), i.e. the sum of the cross-sectional area at 1.3 m above the ground level of all trees on a plot:

(3) $$\text{G}=\pi /4s\overset{n}{\underset{i=1}{\sum }}0.0001*{d_{i}}^{2}$$

where *di* is the diameter (in cm) of the *i-th* tree of the plot and *s* the unit area of a plot (0.1 ha).

• Mean diameter of the tree (Dm, in cm): i.e. the diameter of the tree with mean basal area in the stand was computed for *L. microcarpa* trees as follows:

(4) $$D_{m}=\sqrt{\frac{1}{n}\overset{n}{\underset{i=1}{\sum }}{d_{i}}^{2}}$$

where *d*_*i*_ represents the diameter of the *i*^th^ tree (cm) and *n,* the number of trees found in the plot.

• Lorey's mean height (HL, in meters): i.e. the average height of all trees found in the plot, weighted by their basal area:

(5) $${\text{H}}_{L}=\frac{\sum_{i=1}^{k}g_{i}h_{i}}{\sum_{i=1}^{k}g_{i}}$$

With $g_{i}=\frac{\pi }{4}{d_{i}}^{2}$with *g*_*i*_ the basal area (in m^2^/ha) of *i*^*th*^ tree and *h*_*i*_ the total height (in meters) of *i*^*th*^ tree. This mean height is more stable than an unweighted mean height because it is less affected by mortality and harvesting of the smaller trees and constitutes an important index for woody species management [29].

Tree densities, basal area, mean diameter and Lorey's mean height were compared among phytogeographical zone using separate one-way analysis of variance (ANOVA). The comparison tests were preceded by the Shapiro-Wilk normality test to check the normality of the data. Data that were not normally distributed were normalized using log-transformations. Post hoc analysis of variables showing significant differences was carried out using Tukey's honestly significant difference (HSD) test. All the statistical analyses were performed in R 3.6.1 [30].

#### 2.4.4 Population structure of *Lannea microcarpa*

To characterize the structure of size-class distributions of *L. microcarpa* populations, the observed diameter structures were adjusted to the 3 parameters Weibull theoretical distribution. This approach, using Minitab 16 software, has the advantage to be flexible. Adequacy between the observed distribution and the Weibull distribution was tested with log linear analysis in SAS version 10.2. The Weibull probability density function is defined as follows:

(6) $$f\left(x\right)=\frac{c}{b}{\left(x-a/b\right)}^{c-1}exp\left\{-{\left(x-a/b\right)}^{c}\right\}$$

where *x* stands for diameter of individuals, *a* is the minimum diameter threshold parameter, *b* is the size parameter and *c* represents the shape parameter.

For the regeneration of *L. microcarpa*, the height class structure was established and the demographic trend was analysed following Condit *et al.* [31]. A log-linear regression was performed with the median class and the number of individuals in class plus 1 (ln (Ni+1)). The values of the slopes (a) and the coefficient of determination r^2^ from the regression equations, with their significance level p, were considered to be indicative of the height structure of the species regeneration as suggested by Obiri *et al.* [32]. The height class distribution of regeneration better reflects the difficulties of transition between developmental stages according to environmental conditions [33].

All analyses, except the size-class distributions of *L. microcarpa* populations, were carried out in R software version 3.6.1 [30]. The correlogram was produced in the R package *corrplot*. The Analysis of Variance (ANOVA) was performed with the package *agricolae*. The Minitab software version 14 was used to establish the size-class distributions.

## 3 Results

### 3.1 Woody species associated with *Lannea microcarpa* populations

Natural stands of *L. macrocarpa* were associated with a total of 109 species distributed into 79 genera and 31 families. The most represented families being the Combretaceae (13.11% in the sub-Sahel, 18.31% in the north-Sudanian and 13.97% in the south-Sudanian), the Fabaceae-Mimosoideae (14.75% in the sub-Sahel, 11.27% in the north-Sudanian and 11.28% in the south-Sudanian), the Fabaceae-Caesalpinioideae (9.86% in the north-Sudanian and 9.68% in the south-Sudanian), Rubiaceae (9.8% in the sub-Sahel) and Anarcadiaceae (7.04% in the north-Sudanian).

**Figure 2:**
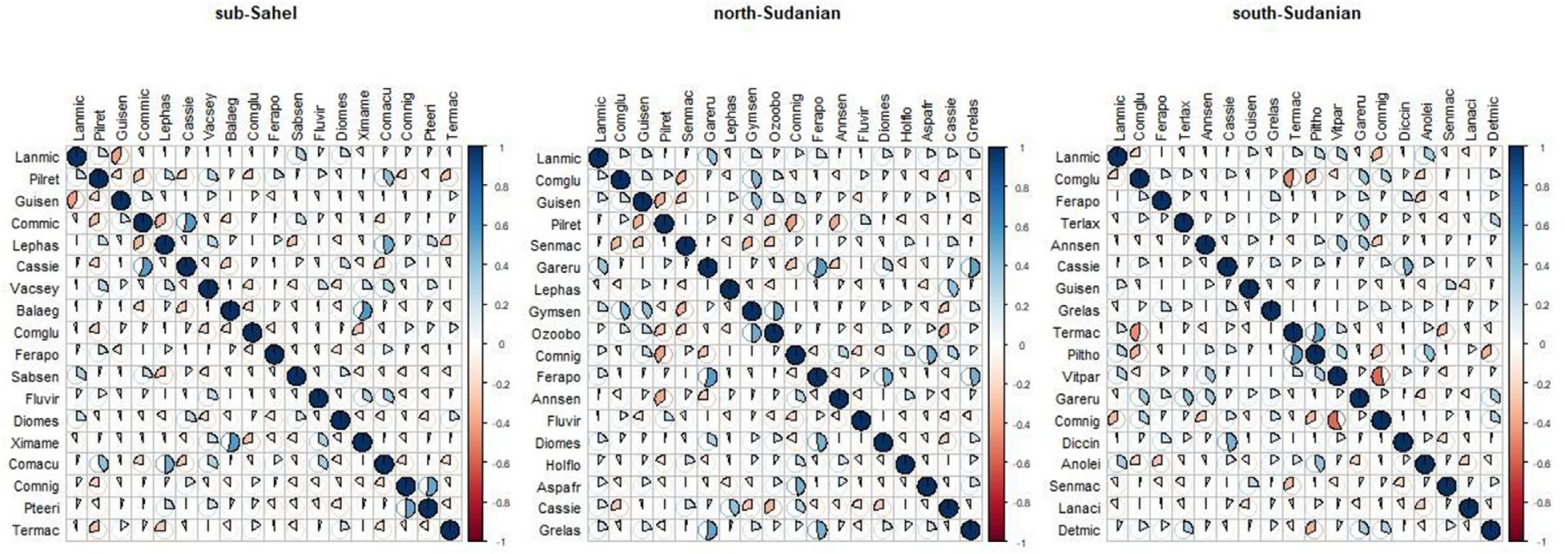
Correlogram showing the frequency with which woody species occurred in the same plot with *Lannea microcarpa* along the phytogeographical zones:in the sub-sahelian, north-Sudanian and south-Sudanian zones (Lanmic = *Lannea microcarpa*; Pilret = *Piliostigma reticulatum*; Guisen = *Guiera senegalensis*; Commic = *Combretum micranthum*; Lephas = *Leptadenia hastata*; Cassie = *Cassia sieberiana*; Vacsey = *Vachellia seyal*; Balaeg = *Balanites aegyptiaca*; Comglu = *Combretum glutinosum*; Ferapo = *Feretia apodanthera*; Sabsen = *Saba senegalensis*; Fluvir = *Flueggea virosa*; Diomes = *Diospyros mespiliformis*; Ximame = *Ximenia americana*; Comacu = *Combretum aculeatum*; Comnig = *Combretum nigricans*; Pteeri = *Pterocarpus erinaceus*; Termac = *Terminalia macroptera*; Senmac = *Senegalia macrostachya*; Gareru = *Gardenia erubescens*; Lephas = *Leptadenia hastata*; Gymsen = *Gymnosporia senegalensis*; Ozoobo = *Ozoroa obovata*; Annsen = *Annona senegalensis*; Holflo = *Holarrhena floribunda*; Aspafr = *Asparagus africanus*; Cassie = *Cassia sieberiana*; Grelas = *Grewia lasiodiscus*; Terlax = *Terminalia laxiflora*; Piltho = *Piliostigma thonningii*; Vitpar = *Vitellaria paradoxa*; Diccin = *Dichrostachys cinerea*; Anolei = *Anogeissus leiocarpa*; Lanaci = *Lannea acida*; Detmic = *Detarium microcarpum*. The blue and pink background colours of the circles in the grid cells indicate positive and negative correlations between the species in proportion to the coloured portion, respectively. For L. microcarpa with the other species, this concerns the first row and column of the grid.

The degree of correlation of woody species with *L. microcarpa* in its natural stands showed by the corrgram revealed that in the sub-Sahel phytogeographical zone, species such as *Saba senegalensis* (A.DC.) Pichon, *Piliostigma reticulatum* (DC.) Hochst and *Diospyros mespiliformis* Hochst. ex A.DC. are positively correlated with à *L. microcarpa* (Figure 2). In the north-Sudanian zone, *L. microcarpa* is positively correlated with *Gardenia erubescens* Stapf & Hutch., *Gymnosporia senegalensis* (Lam.) Loes., *Feretia apodanthera* Delile and *Grewia lasiodiscus* K.Schum (Figure 2). In the south-Sudanian zone, species that are positively correlated with *L. microcarpa* are *Anogeissus leiocarpa* (DC.) Guill. & Perr., *Piliostigma thonningii* (Schumach.) Milne-Redh., and *Vitellaria paradoxa* C.F. Gaertn., (Figure 2).

### 3.2 Diversity parameters of *Lannea microcarpa* habitats

The calculated species richness (df = 2, F = 64.12 and p < 0.001), Shannon's diversity index (df = 2, F = 97.6 and p < 0.001) and Evenness of Pielou index (df = 2, F = 53.67 and p < 0.001) vary significantly between the phytogeographical zones (Table 2). Among the three phytogeographical zones, the highest diversity is observed in the south-Sudanian one (H’ = 2.74 ± 0.32), with a total number of 93 species. This zone also exhibits a good distribution of species (E = 0.84 ± 0.04). The sub-Sahelian zone had the lowest diversity (H’ = 1.87 ± 0.30), with a total number of 61 species and a mean species richness of 14.25 ± 3.73.

**Table 2:** Diversity indices of *Lannea microcarpa* stands (Mean ± standard deviation).

Phytogeographical zones	Family	Genera	Species richness	MSR	*H′*	*E*
Sub-Sahelian	22	44	61	14.25 ± 3.73^a^	1.87 ± 0.30^a^	0.71 ± 0.07^a^
North-Sudanian	23	50	71	19.10 ± 4.75^b^	2.15 ± 0.28^b^	0.74 ± 0.06^a^
South-Sudanian	31	69	93	27.00 ± 7.32^c^	2.74 ± 0.32^c^	0.84 ± 0.04^b^

MSR = Mean species richness, H’ = Shannon's diversity index, E = Evenness of Pielou index. In each column, values with different letters are significantly different according to the 5% Tukey test.

Jaccard's similarity index revealed a low to a medium similarity (>50%) between the different habitats of *L. microcarpa* across the three phytogeographical zones (Table 3). The highest similarity (71%) was observed in the habitats located in the north-Sudanian and sub-Sahelian zones.

**Table 3:** Jaccard's similarity of *Lannea microcarpa* stands according to phytogeographical zones.

	Sub-Sahelian	North-Sudanian	South-Sudanian
Sub-Sahelian	1		
North-Sudanian	0.71	1	
South-Sudanian	0.45	0.55	1

### 3.3 Spatial distribution and demographic characteristics of *Lannea microcarpa*

The calculated values of the GI are 0.03 for all three phytogeographical zones. These GI values are low and close to zero, reflecting a random distribution pattern of the species.

Analysis of variance revealed that latitudinal gradient had a highly significant effect on mean densities of adult (F = 18.78, p < 0.001) and juvenile (F = 7.20, p < 0.001) individuals, mean diameter (F = 81.16, p < 0.001) and Lorey's mean height (F = 28.13, p < 0.001) of *L. macrocarpa* (Table 4). Basal area did not vary significantly along the latitudinal gradient (p > 0.05). The highest value of the mean density of adult trees was found in the north-Sudanian zone whereas the highest values of the mean densities of the juvenile individuals was in the south-Sudanian zone (Table 4).

**Table 4:** Structural parameters (mean ± standard deviation) of *Lannea microcarpa* trees and regeneration in the three phytogeographical zones.

Parameters	Sub-Sahelian	North-Sudanian	South-Sudanian	*p*-values
**Trees**				
Mean density (ind/ha)	54.44 ± 22.11^**b**^	93.11 ± 36.73^**a**^	89.11 ± 37.58^**a**^	<0.001
Mean diameter (cm)	38.70 ± 15.88^**a**^	26.05 ± 11.20^**c**^	28.30 ± 12.06^**b**^	<0.001
Mean basal area (m^2^/ha)	7.5 ± 3.73^**a**^	5.88 ± 2.87^**a**^	6.62 ± 3.39^**a**^	0.071
Lorey's mean height (m)	8.27 ± 1.5^**a**^	6.59 ± 1.24^**b**^	6.43 ± 0.94^**b**^	<0.001
**Regeneration**				
Mean density	471.11 ± 1202.38^a^	2764.44 ± 4416.53^ab^	4480.00 ± 7404.00^b^	<0.001

In each line values with different letters are significantly different according to the 5% Tukey test.

### 3.4 Population structure of *Lannea microcarpa*

**Figure 3:**
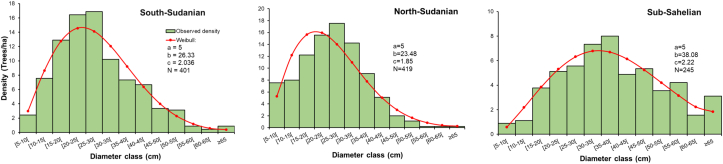
Stem diameter class distribution of *Lannea microcarpa* trees per phytogeographical zone.

The diameter class distribution of *L.microcarpa* trees in the three phytogeographical zones showed an irregular distribution of individuals with a predominance of trees of intermediate classes compared to those of extreme classes (Figure 3). In fact, individuals having a diameter below 15 cm are poorly represented compared to individuals of diameter classes between 20 and 40 cm which represent more than 50% of the population. In addition, the values of the shape parameter "*c*” are between 1 and 2.23, indicating a straight asymmetric distribution that reflects a population with low regeneration potential and experiencing difficulties in the succession of different growth stages. The observed distribution in the phytogeographical zones fits well with the theoretical Weibull distribution (p > 0.05) according to the log-linear analysis.

The values of the slopes of the linear regression equation for regeneration are negative in all phytogeographical zones, revealing the dominance of individuals with low height (Table 5). The *r*^*2*^ and *p*-values illustrate that there is a good demographic trend in the north and south-Sudanian zones compared to the sub-Sahelian one.

**Table 5:** Regression slope values of height class distributions of *Lannea microcarpa* regeneration per phytogeographical zone.

Phytogeographical zones	Slope	*r*^*2*^	*p-values*
Sub-Sahelian	-1.22	0.38	0.266
North-Sudanian	-2.11	0.96	0.003∗
South-Sudanian	-3.49	0.86	0.021∗

**Figure 4:**
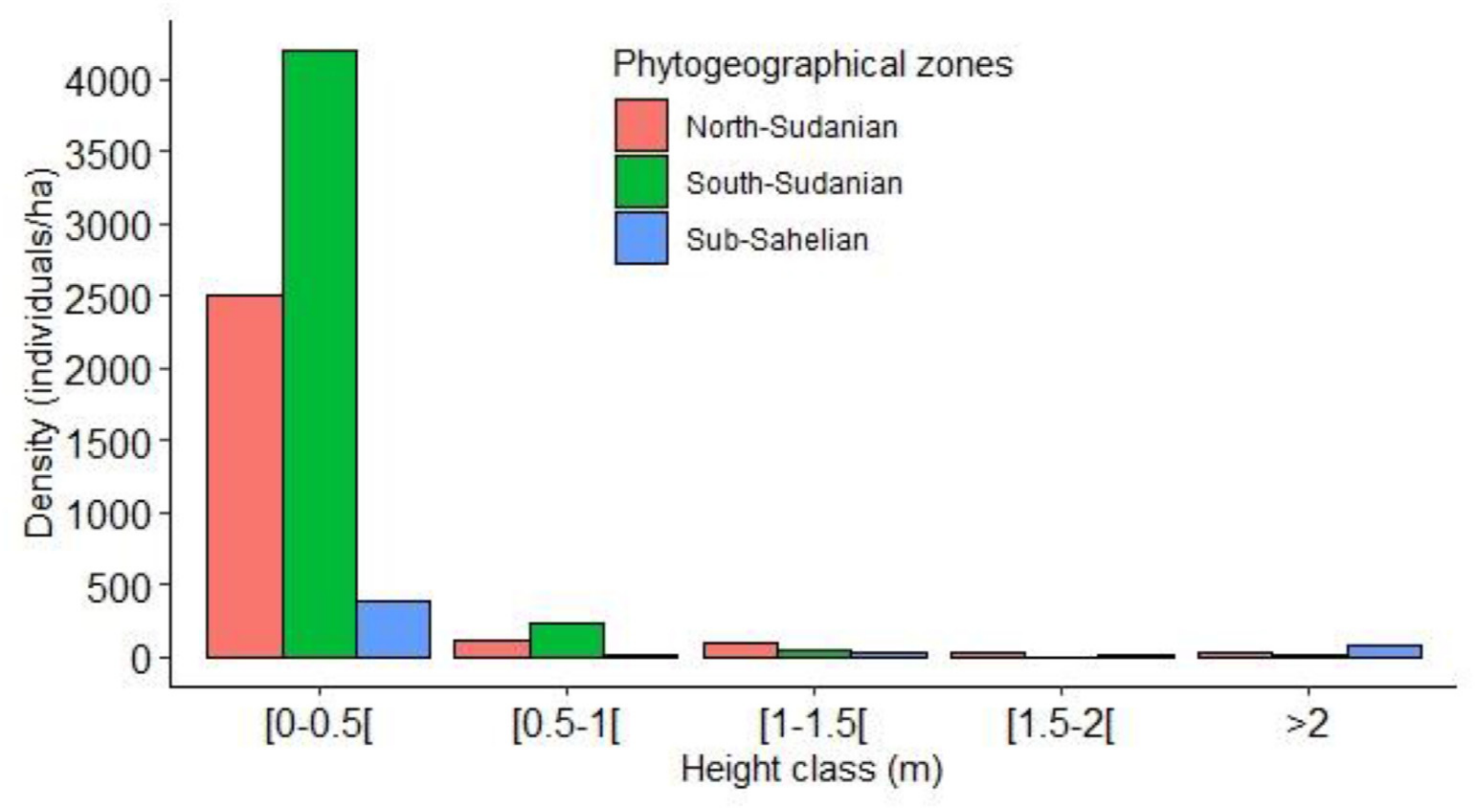
Height class distribution of juveniles of *L. microcarpa* in the three phytogeographical zones. The heights of the juveniles were grouped into five height classes at 0.5 cm intervals: [0–0.5[ = class 1, [0.5–1[ = class 2, [1–1.5[ = class 3, [1.5–2[ = class 4, height >2 m = class 5.

As for the analysis of the distribution of height classes (Figure 4), it showed a decrease in the number of individuals from the lowest to the highest classes, often with the absence of certain classes in some zones. This reveals unsuccessful transition juveniles from the lower classes to the higher classes, reflecting difficulties of survival in juvenile individuals.

## 4 Discussion

### 4.1 Floristic diversity and species associated with *Lannea microcarpa*

Overall, 109 woody species were recorded in *L. microcarpa* stands. This species richness represents 20.52 % of the woody flora of Burkina, with reference to Thiombiano *et al.* [34]. Indeed, Thiombiano *et al.* [34] reported that the woody flora of Burkina Faso is made up of 531 species. This number of species is higher than that recorded by Bognounou *et al.* [35] in the four phytogeographical zones of the country (74 species). This difference could be explained by the different sampling methods used, the plant communities studied and the objective of the study. The dominance of Combretaceae and Fabaceae-Mimosoideae families which characterize dry climates, reveals the savanna character of these plant communities associated with anthropogenic pressures and climate pejoration [26].

The results of the correlogram show that the woody species that are indicators of the presence of *L. microcarpa* in the environments differ from one zone to another one. This can be explained by the high ecological plasticity of the species. *L. microcarpa* adapts to a variety of environmental conditions: deep soils of plains, lowlands, hill bottoms and temporarily flooded plains [15,24]. In order, these species would adapt to ecological conditions similar to those of *L. microcarpa*, which could be a basis for a possible silviculture of the species.

In terms of ecological parameters, the increase in mean values of species richness and Shannon index from the sub-Sahel to the south-Sudanian is consistent with previous studies that indicate a progressive increase in species diversity from the north to the south in most parts of Burkina Faso [36]. This could be due to the more severe climatic conditions in the northern areas, making the plants more vulnerable to human disturbance [37,38]. This gradient correlates positively with increasing rainfall. The differences in species composition between sites could be due to microsite factors (i.e. abiotic and biotic factors). The high values of the evenness index of Pielou (E) indicate that the dominant species have a relatively homogeneous distribution. According to Glèlè Kakaï and Sinsin [29], the low value of evenness index of Pielou reveals the dominance of at least one species in the stand. Jaccard's index values showed the greater or lesser similarity in species composition in *L. microcarpa* plant formations along the different zones. The low values of the similarity index indicate a high β-diversity between populations and could be related to the different environmental and habitat conditions across the three phytogeographical zones. Thus, the pattern of diversity and distribution of taxa highlights the importance of the phytogeographical gradient as an influential factor in the flora.

### 4.2 Spatial distribution and dendrometric characteristics of *Lannea microcarpa*

The random distribution of *L. microcarpa* across the three phytogeographical zones could be explained by the fact that the seminal pathway seems to be its preferred way of multiplication [14]. Unfortunately, the efficiency of seminal regeneration is compromised due to climatic pejoration and human pressures [39].

Regarding the stand structure of *L. microcarpa*, our results show that the phytogeographic gradient has a significant influence on the variables, except for the basal area. The high densities of trees in both north-Sudanian and south-Sudanian zones can be explained by the better rainfall in these areas which could also influence soil moisture and nutrient cycling, thus affecting the quality of resources available for plant growth [40]. The slight increase in density in north-Sudanian zone could be related to the autoecology of the species that probably prefers intermediate environment between semi-arid sub-Sahel and sub-humid south-Sudanian zones. This finding could also be explained by the fact that *L. microcarpa* is a species that presents very high local densities in suitable environments [41]. Our results are consistent with those of Thiombiano et al. [42] who found a high density of *L. microcarpa* (122 ± 101 individuals/ha) in Nobéré, a locality in the north-Sudanian zone which seems to be the area where the species finds an ecological optimum. The low density of trees and juveniles associated with high values of mean diameter and mean height in the sub-Sahelian are the consequences of disturbances such as bushfires, overgrazing and repeated droughts as reported by Kabré *et al.* [43].

### 4.3 Population structure of *Lannea microcarpa*

The diameter class distribution of *L. microcarpa* reveals unstable populations in all the three phytogeographical zones. The instability is expressed by the predominance of trees of intermediate diameter classes and scarcity of individuals of small diameter classes. This same trend was found by Haarmeyer et al. [11] and could be explained by the pressure on smallest diameter classes individuals during construction works due to their ease of transport as reported by other authors for other species [32]. However, it should be noted that this instability is much more marked in the sub-Sahel with a near absence of individuals in the [5–10[ diameter class. In this phytogeographical zone, juveniles are particularly affected by bush fires, overgrazing and climatic deterioration [34,44]. According to Bognounou *et al.* [35]^,^ latitudinal gradients are ultimately dependent on the historical, geographic, biotic, abiotic and stochastic forces affecting the geometry, internal structure and location of species ranges in ecological or evolutionary time.

With regard to the regeneration, although the slopes of the regression equations are all negative showing the predominance of individuals of class 1, the species shows difficulties in recruitment and transition between the different classes in its range. *L. microcarpa* is known to have a very low germination rate due to the high oil content of seeds [45], which causes them to lose viability quickly, and also due to their tegument dormancy [14]. In addition, the almost complete harvesting of fruits in some localities for consumption and trade during the lean season may also explain the low natural recruitment [42]. A great disparity between the different classes of height is noticeable in the three phytogeographical zones. Indeed, factors such as droughts, the long dry season, bush fires, overgrazing, agricultural clearing and excessive harvesting affect the natural regeneration of woody plants [46]. These limiting factors compromise particularly the successful renewal of *L. microcarpa* populations.

## 5 Conclusion

This study investigated the variation in the populations structure of *Lannea microcarpa* and associated woody species diversity along the latitudinal gradient in Burkina Faso. The results showed significant influence of the latitude on the demographic parameters of *L. microcarpa* and woody flora diversity of stands. Diameter classes distribution of *L. microcarpa* revealed populations dominated by trees of intermediate diameters across the study area. Such observations indicate unstable population structures of the species, characterized by the absence of some diameter classes in all three phytogeographical zones. The mean density of trees in stands is above 50 individuals/ha, indicating that *L. microcarpa* has a seed-bearing potential that can ensure its sustainability, but controlled use is necessary for its conservation. Evidence is also provided that the species experiences difficulties in recruitment and transition between the different height classes in the study sites. These results call for measures to monitor germination, survival and growth of seedlings and to protect mature trees of the species. For example, given the vulnerable status of the species, it would be interesting to consider conservation strategies that involve local populations and sensitize them to the need to adopt assisted natural regeneration (ANR) in agroforestry systems. To better understand the influence of land use patterns on the demographic characteristics of the species, it would be crucial to study in depth the human disturbance regimes on its population structure.

## Declarations

### Author contribution statement

Béatrice Tinguéri, Kangbéni Dimobe and Benjamin Lankoandé: Conceived and designed the experiments; Performed the experiments; Analyzed and interpreted the data; Wrote the paper.

Amadé Ouédraogo and Joseph Issaka Boussim: Conceived and designed the experiments; Contributed reagents, materials, analysis tools or data; Wrote the paper.

### Funding statement

Béatrice Tinguéri was supported by 10.13039/501100011054Danish International Development Agency [No. 10-002 AU] and International Foundation for Science [No. D/5647-1].

### Data availability statement

Data will be made available on request.

### Declaration of interest’s statement

The authors declare no conflict of interest.

### Additional information

No additional information is available for this paper.
